# The boomerang effect of zero pricing: when and why a zero price is less effective than a low price for enhancing consumer demand

**DOI:** 10.1007/s11747-022-00842-1

**Published:** 2022-02-14

**Authors:** Xiaomeng Fan, Fengyan Cindy Cai, Galen V. Bodenhausen

**Affiliations:** 1 School of Entrepreneurship and Management, ShanghaiTech University, Shanghai, 201210 China; 2 Antai College of Economics and Management, Shanghai Jiao Tong University, Shanghai, 200030 China; 3 Kellogg School of Management and Department of Psychology, Northwestern University, 2029 Sheridan Road, Evanston, IL 60208 USA

**Keywords:** Zero price, Low price, Incidental costs, Cognitive scrutiny, Field study

## Abstract

Prior literature has demonstrated the power of zero pricing to boost consumer demand, but the current research shows a novel “boomerang effect”: a zero (vs. low, nonzero) price can lower demand when the offer comes with high incidental costs (e.g., the time cost in commuting to an offline class; the physical risk of getting a new vaccine). Five studies show that zero pricing, relative to low pricing, has a boosting (boomerang) effect on demand when incidental costs are low (high). The diverging effects of zero pricing on demand are explained by a dual-process model with a positive affective pathway and negative scrutiny pathway. Zero pricing triggers both positive affect and cognitive scrutiny of incidental costs; when incidental costs are high, the scrutiny pathway overrides the affective pathway and decreases demand. The finding has managerial implications as incidental costs often vary widely between marketing channels and over a product’s life cycle.

**Supplementary Information:**

The online version contains supplementary material available at 10.1007/s11747-022-00842-1.

## Introduction

Companies often use a penetration pricing strategy when introducing new products to a market. The penetration strategy involves offering a product at a low price to generate immediate demand for the new offering and gain a significant market share (Dean, 1976; Tellis, 1986). Following the law of demand, lower prices are more attractive to consumers, so a zero price ostensibly should generate the highest demand. Extant literature has demonstrated that zero prices, compared to low, nonzero prices, can significantly increase demand for a product (Baumbach, 2016; Nicolau & Sellers, 2012; Shampanier et al., 2007). The *boosting effect* of zero pricing on consumer demand is driven primarily by consumers’ positive affect, which is markedly enhanced when the price is zero (vs. low, nonzero; Shampanier et al., 2007). Consumers’ positive reactions extend to pseudo-free offers—those that are presented as free but require certain concessions (e.g., completing a customer survey, providing personal information, or watching an ad; Dallas & Morwitz, 2018).

The present research investigates whether there are conditions in which a zero price is *less* effective than a nonzero price at penetrating the market. If a zero price results in lower demand than a nonzero price, this would constitute a *boomerang effect*—that is, although companies intend to drive demand up by reducing the price to zero, the strategy produces the opposite effect. We propose that the effect of zero (vs. low, nonzero) pricing on consumer demand depends on the incidental costs: nonmonetary costs that are inherently involved in acquiring or using the product (e.g., the time required to commute to the store and/or wait in line). Consumers bear the same incidental costs regardless of the product’s monetary price.

We use both field and laboratory data to show that a zero (vs. low, nonzero) price can drive down consumer demand for a product (a *boomerang effect*) when incidental costs are high. When incidental costs are low, a zero price boosts consumer demand (a *boosting effect*), as has been demonstrated in the literature. The boomerang and boosting effects occur because zero pricing can trigger both positive affect and close scrutiny of incidental costs; the affective process dominates when incidental costs are low, while the scrutiny process dominates when incidental costs are high. A low, nonzero price is also appealing, but it generates much less scrutiny than a zero price, so a low-priced offer remains attractive when incidental costs are high.

The present research contributes to the existing literature in three noteworthy ways. First, no previous research has shown in a single study that zero pricing (relative to low pricing) can both facilitate and inhibit consumer demand for a product during a promotion. As reviewed in Table 1, all prior research focused on either the positive or negative effects of zero pricing without developing a framework that explains both outcomes. The present research documents both a positive and a negative effect and develops an integrative theoretical model—a dual-process model with competing affective and scrutiny pathways—that explains when and why zero pricing causes each effect. Second, the present research offers insight into the understudied topic of incidental costs in consumer decision-making. Incidental costs accompany almost all purchases in daily shopping, but their impacts on consumer behavior have drawn little attention from researchers, perhaps because incidental costs are not charged by the sellers and usually are not very salient to consumers. We find that a zero price is distinct from other promotional prices in that it triggers the *cognitive scrutiny* of incidental costs; when the incidental costs are high, scrutiny drives down consumer demand. Finally, for marketing practice, incidental costs represent a novel angle from which to understand marketing mix variables (e.g., channels: online vs. offline stores; products: newly developed vs. mature). Our findings may enable companies to make more strategic choices about discount prices, particularly in omni-channel marketing and when adapting promotion strategies throughout the product life cycle.

**Table 1:** Literature review: the pros and cons of zero pricing

Paper	Product context	IV	DV	Effect	Key findings	Mechanism tested/implied	Theoretically-relevant moderator
Shampanier et al. (2007)	Single	Zero price vs. low price	Consumer demand for the promoted product	Positive	The zero-price effect: Zero pricing (vs. low pricing) significantly increased demand for the product.	A zero (vs. low) price evokes a more positive affect.	Rational (vs. affective) evaluation
Hossain & Saini (2015)	Single	Zero price vs. low price	Consumer demand for the promoted product	Positive	The zero-price effect was stronger for hedonic than for utilitarian products.		Hedonic (vs. utilitarian) products
Dallas & Morwitz (2018)	Single	Pseudo-free vs. not free	Consumer demand for the promoted product	Positive	Consumers were more likely to accept pseudo-free offers (with a non-monetary cost such as completing a customer satisfaction survey) than non-free offers without nonmonetary costs.	Consumers generate neutral or positive attributions when firms make pseudo-free offers, and these attributions lead consumers to perceive the offers as fair.	Factors that increase negative attributions
Hüttel et al. (2018).	Single	Pseudo-free vs. not free	Consumer demand for the promoted product	Positive	Consumers perceived nonmonetary costs (e.g., watching ads for streaming services) to be lower in a pseudo-free offer than in a paid offer, thus increasing their likelihood of taking the offer.	Free offers elicit positive affect and activate the social norm of reciprocity.	N/A
Nicolau & Sellers (2012)	Multi-component	Better product without a free item vs. regular product with a free item	Consumer demand for the promoted product	Positive	The zero-price effect occurred with multi-component products.	A zero (vs. low) price evokes a more positive affect.	N/A
Baumbach (2016)	Multi-Component; Single	Zero price vs. low price	Consumer demand for the promoted product	Positive	The zero-price effect occurred with both single and multi-component products, but the effect was weaker when the context involved high-priced products (vs. low-priced products).		N/A
Ma et al. (2018)	Multi-component	Supplemental item: normal discounted price, low discounted price, vs. zero price	Consumer demand for the promoted product; neural response	Positive	A free supplemental item led to the largest LPP amplitude, which indicates that the free offer induced a more positive affect.		N/A
Chandran & Morwitz (2006)	Multi-component	Free promotion vs. monetary price promotion	Sensitivity toward negative information related to quality	Positive	A free signal in a purchase (e.g., free shipping) reduced consumers’ sensitivity to negative quality information about the focal product.	The free element is highly salient, so consumers focus on it more than on the focal product.	Ease of recalling reasons to buy
Palmeira & Srivastava (2013)	Multi-component	Zero price vs. low price	WTP for the product after promotion	Positive	When a product was promoted at a zero (vs. low) price, consumers were willing to pay more for the product when the promotion ended.	Consumers anchor on the low price of the supplemental product to estimate its value. For a zero-priced item, however, consumers use the price of the focal product, which usually is high.	The price of the focal product; the presence of a new price anchor
Raghubir (2004)	Multi-component	Seeing (vs. not seeing) a free gift ad before encountering a target product in the same category as the gift	WTP and purchase intention for the target product	Negative	Both the WTP and the purchase intention for the target product (e.g., branded pearl sets) were lower if consumers previously saw a similar product (e.g., a pearl bracelet) promoted as a free gift.	The “value-discounting hypothesis”: consumers infer that a product offered for free is of low value. Such an inference may influence consumers’ valuation of similar products.	N/A
Kamins et al. (2009)	Multi-component	A mention of free vs. no mention of free for the supplemental product in a bundle	WTP for each product and for the bundle after the promotion	Negative	When a supplemental product was mentioned (vs. not mentioned) as free, WTP decreased for both the supplemental product and the focal product (when sold individually).	An extension of the value-discounting hypothesis: consumers infer that when a bundle contains a free supplemental product, all products in the bundle are of low value.	Cognitive load
Mao (2016)	Multi-component	Zero price vs. low price for upgrading	Consumer demand for the promoted product	Negative	An upgrade offer was less attractive with a zero price (e.g., upgrade for free) than with a token price (e.g., for 1 cent).	A zero price, but not a low, nonzero price, inhibits the comparison against the regular price.	Articulating the savings before (vs. after) evaluations
Thepresent research	Single	Zeroprice vs. low price	Consumer demand for the promoted product	Positive and Negative	Zero pricing (relative to low pricing) boosts demand when incidental costs are low but hurts demand when incidental costs are high (i.e., a boomerang effect).	A dual-process model: a zero (vs. low, nonzero) price consistently elicits more positive affect, but it also triggers more cognitive scrutiny of incidental costs.	Incidentalcosts

## Literature review: the pros and cons of zero pricing

As a special promotional price, the zero price has drawn great interest in from marketing researchers. The existing literature concerns the type of product offer (single vs. multi-component product), the nature of the free offer (truly free vs. pseudo-free), and the nature of the comparison offer, as well as the results and theoretical mechanisms (see Table 1).

Almost all extant findings indicate that a zero price outperforms other promotional prices on various measures, including the demand for the promoted product (e.g., Ma et al., 2018; Nicolau & Sellers, 2012; Shampanier et al., 2007) and the willingness to pay (WTP) for the product after the promotion ends (e.g., Palmeira & Srivastava, 2013). The benefits of a zero price can be elicited by both truly free offers (e.g., Baumbach, 2016; Hossain & Saini, 2015; Palmeira & Srivastava, 2013; Shampanier et al., 2007) and pseudo-free offers (Dallas & Morwitz, 2018; Hüttel et al., 2018), and with both single products (Hossain & Saini, 2015; Shampanier et al., 2007) and multi-component products (e.g., Chandran & Morwitz, 2006; Ma et al., 2018; Nicolau & Sellers, 2012; Palmeira & Srivastava, 2013).

Three papers provide exceptions to the general rule that zero pricing boosts consumer demand. Raghubir (2004) and Kamins et al. (2009) tested a value-discounting hypothesis; consumers infer that a discounted (or free) product has a low production cost, so consumers lower their WTP for the product once the promotion ends. A noteworthy study by Mao (2016) showed that a zero price (but not a low, nonzero price) inhibits comparisons against the regular price, thus reducing the attractiveness of the offer. The present research augments our understanding of the potentially adverse effects of zero pricing by identifying a novel psychological mechanism that differs from both the value-discounting hypothesis and Mao’s study in two important ways.

First, while the value-discounting hypothesis focuses on WTP for the promoted product *after* the promotion ends, we consider the attractiveness of the promoted product *during* the promotion. Moreover, the research on the value-discounting hypothesis did not provide empirical evidence that zero pricing is less effective than low, nonzero pricing at increasing demand. Instead, Palmeira and Srivastava (2013) showed that a zero (vs. low, nonzero) price leads to *higher* perceived product value, so our boomerang effect of a zero (vs. low, nonzero) price on consumer demand cannot readily be explained by value-discounting.

Second, Mao’s study showed that a zero price has a special ability to inhibit comparisons against the regular price—in other words, a comparison-inhibiting mechanism. Specifically, Mao found that consumers who were offered a free upgrade did not spontaneously compare the zero price with the original price and thus were less likely to appreciate the substantial savings than those who were offered a low, nonzero price. Our proposed scrutiny mechanism is distinct from the comparison-inhibiting mechanism, so our findings complement Mao’s by illuminating a novel reason for the unintended consequences of zero pricing. Together, the findings offer a more comprehensive picture of the unique psychological attributes of a zero price.

In the next section, we explain the key concept of incidental costs and the scrutiny mechanism behind the boomerang effect of zero pricing.

## Monetary price, nonmonetary price, and incidental costs

A *price* is the quantity of payment or compensation given by a customer to a seller in exchange for a product (Schindler, 2011). Usually, the price is paid in the local currency (e.g., one gallon of spring water for $1.87), but sometimes, sellers request nonmonetary forms of payment. For example, a seller might ask a customer to complete a customer satisfaction survey because customer feedback is an asset for the business. Other examples of nonmonetary prices include watching an advertisement to obtain free WiFi at the airport and providing personal information such as a phone number and home address to obtain a free product. These offers can be thought of as “pseudo-free” because they do not require money but nevertheless provide a benefit to the seller.

### Incidental costs

Incidental costs are nonmonetary costs borne by the consumer, and they accompany almost all purchases. For example, students who wish to take offline classes need to spend time and effort to travel to the classroom. Incidental costs have three important differences from the nonmonetary prices in pseudo-free offers. First, the consumer must bear the same incidental costs regardless of the price (zero or nonzero) asked by the seller. For example, a student will incur the same transportation costs whether or not an offline class is free. Second, incidental costs are not set by the seller, and they do not benefit the seller. Third, incidental costs are generally inevitable and inherent to the purchase, so they typically are not mentioned explicitly in an offer. Instead, incidental cost information is implicit and must be detected by consumers themselves. In many circumstances, the implicit incidental costs may receive little if any consideration as consumers focus primarily on the product and its monetary price. This research proposes that when an offer involves a zero monetary price, consumers scrutinize the incidental costs more than they would otherwise.

### Categories of incidental costs

Murphy and Enis (1986) proposed that nonmonetary costs can be categorized along two dimensions: *effort* and *risk*. *Nonmonetary effort* includes the time required to travel, shop, and wait before consumers can obtain or use the product. There are four types of *nonmonetary risks*: functional risk (i.e., the product might not perform as expected), physical risk (i.e., product use might jeopardize the customer’s or others’ safety), social risk (i.e., the product choice might cause embarrassment), and psychological risk (i.e., the product choice might harm the customer’s ego). These categories are widely accepted in the marketing literature (e.g., Jacoby & Kaplan, 1972; Kotler & Keller, 2016). The present research considers both types of nonmonetary costs: effort in Studies 1–4 and risk in Study 5.

All types of risk entail uncertainty about experiencing adverse effects (Holton, 2004). Existing literature clearly demonstrates that uncertainty is emotionally costly (for a review, see Anderson et al., 2019). Greco and Roger (2003) review evidence that “uncertainty constitutes a powerful stressor” (p. 1057), and Peters et al. (2017) call uncertainty the “essence of stress” (p. 164). Other research shows that uncertainty appraisals are strongly linked to negative emotions like anxiety and fear (Carleton, 2016; So et al., 2015). Given the clear relationship between uncertainty and negative emotions, it is reasonable to treat the emotional cost associated with risk as a type of nonmonetary cost.

## Zero pricing enhances cognitive scrutiny of incidental costs

Incidental costs influence purchase decisions only when the consumer takes the time and effort to consider them. A large literature has shown that cognitive scrutiny of this sort occurs only when people have the motivation and opportunity to engage in cognitive elaboration (for a review, see Fazio & Olson, 2014). We expect zero pricing to increase both consumers’ motivation and opportunity to scrutinize incidental costs.

Consumers may be more motivated to scrutinize an offer with a zero (vs. low, nonzero) price because a zero price is surprising—it seems to violate market transaction norms (e.g., consumers usually need to pay something to obtain a product from the seller). Prior research shows that consumers engage in more cognitive elaboration when an event is surprising (vs. not surprising; e.g., Leuker et al., 2019; Sujan et al., 1986).

The increase in scrutiny is likely to be focused on incidental costs rather than the zero price itself. Consumers receiving a zero-priced (vs. low-priced) offer do not need to think about the affordability of the offer at all; thus, they have more opportunity to scrutinize the incidental costs, which otherwise tend to be neglected. Our argument is based on prior evidence that explicit monetary information serves as the base price in consumer deliberations, and consumers process explicit price information before considering implicit incidental costs. For example, Morwitz et al. (1998) found that partitioning prices into a base price and a surcharge (e.g., a shipping fee) decreased the total cost recalled by consumers, likely because consumers paid more attention to and anchored on the base price and then adjusted insufficiently for the surcharge, which often is ignored (much like incidental costs). Castilla and Haab (2012) confirmed that people allocate attention to information regarding the monetary price of a task while neglecting incidental costs associated with the task.

People have limited attentional resources (DellaVigna, 2009), so the allocation of attention to the evaluation of affordability necessarily reduces the cognitive resources available to scrutinize other aspects such as incidental costs. We argue that consumers allocate most of their attention to affordability even when the price is low (but nonzero). Consumers are likely to evaluate whether the deal is worth the requested amount of money, and they must consider practical matters regarding the payment transaction (e.g., Should I use cash, credit, or debit card? Do I have a credit card handy?). When the base price is zero, however, consumers do not need to spend any time or effort evaluating affordability or transaction logistics, so they can allocate all their attentional resources to the incidental costs.

Taken together, we propose that a zero (vs. low) price affords consumers more motivation and opportunity to scrutinize the incidental costs associated with the offer. Then, a zero (vs. low) price should decrease consumers’ acceptance of an offer if the incidental costs are high, but not if they are low. In the next section, we articulate a dual-process model to conceptualize consumers’ reactions toward zero prices.

## A dual-process model of the diverging effects of zero pricing on demand

The present research proposes that consumers’ responses toward zero prices are best understood with a dual-process model: zero prices (relative to low, nonzero prices) generate more positive affect and enhance the scrutiny of incidental costs.

**Fig. 1:**
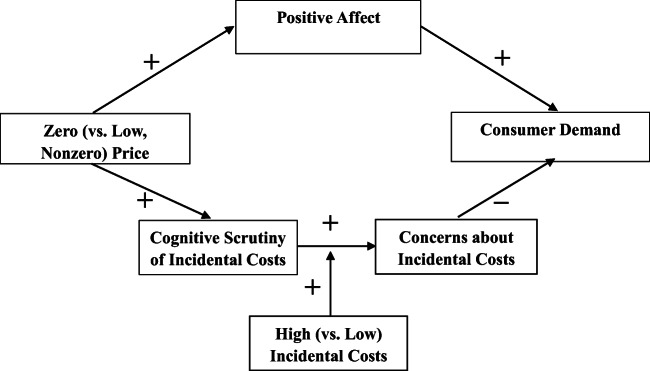
Conceptual model

The affective process is well established in the literature; a zero price makes consumers happier than a low price, and the increase in happiness leads to increased demand for the product (Shampanier et al., 2007). In this research, we propose that the affective process dominates only when incidental costs are low, in which case additional scrutiny will not make the product unattractive. When incidental costs are high, however, then close scrutiny should lead to concerns about the drawbacks of the offer, thus overriding the affective pathway. In short, when incidental costs are low, the dominant affective pathway leads to a positive effect of zero pricing on demand (i.e., the boosting effect), but when incidental costs are high, the dominant scrutiny pathway leads to a negative effect of zero pricing on demand (i.e., the boomerang effect). See Fig. 1 for the proposed model.

The psychological literature has identified many kinds of dual-process models. The one proposed in the current research is in the “parallel-competitive” class (Sloman, 1996; for a review see Evans & Stanovich, 2013), in which the two processes are postulated not to interact but rather to compete for control over the response. Some past research indicates that positive affect can reduce cognitive elaboration but only *in the absence of competing goals and motivation*s (e.g., Bodenhausen et al., 1994; Forgas, 1989). In the present context, zero pricing activates a competing goal: to understand whether the offer is “too good to be true.” For this reason, the current research does not hypothesize any effect of positive affect on the scrutiny process. Instead, affect independently increases demand while scrutiny independently increases attention to incidental costs; when incidental costs are high, the associated concerns can overwhelm the affective pathway. Specifically, we hypothesize:

### H1a

(the boosting effect) Zero (vs. low, nonzero) prices increase consumer demand when incidental costs are low.

### H1b

(the boomerang effect) Zero (vs. low, nonzero) prices decrease consumer demand when incidental costs are high.

Regarding the mechanism, we hypothesize:

### H2a

Zero (vs. low, nonzero) prices generate more positive affect.

### H2b

Zero (vs. low, nonzero) prices elicit greater scrutiny of incidental costs.

### H2c

The boosting effect of zero (vs. low, nonzero) pricing on demand in the low-incidental-cost condition is mediated by heightened positive affect.

### H2d

The boomerang effect of zero (vs. low, nonzero) pricing on demand in the high-incidental-cost condition is mediated by increased concerns about incidental costs.

## Empirical overview

Five studies tested the hypotheses. In Study 1, we used real-world data from a large education company that offers after-school tutoring for K-12 students in both online and offline formats. We found that the relationship between the price level and consumer demand was linear and negative for online classes (with low incidental costs) but had an inverted U-shape for offline classes (with high incidental costs such as the commute time). Thus, we found a boomerang (boosting) effect of zero pricing on consumer demand when incidental costs were high (low). In Study 2, we used a controlled lab environment to test the mechanism behind the findings in Study 1. The results support our theoretical model: the boosting effect of a zero (vs. low, nonzero) price on consumer demand for an online class is driven by greater positive affect in response to the zero price, while the boomerang effect of the zero (vs. low, nonzero) price on consumer demand for an offline class is driven by an increase in concerns about the time cost involved.

In Study 3, we showed that a high cognitive load can lead to the boosting effect even when incidental costs are high, providing additional evidence for the scrutiny mechanism and against alternative accounts involving an automatic association between zero prices and negative concepts (e.g., bad quality). Study 4 used the same product (an online seminar) for all participants and took advantage of natural variation in the incidental costs between participants; Study 4 also used an extremely low price (1 cent) to confirm that the boomerang effect is special to zero pricing. Finally, Study 5 showed that the boomerang effect occurs when incidental costs involve risk rather than effort.

In Studies 2, 4, and 5, we examined the interaction between the price level and incidental costs in an additional way, which we report in Web Appendix A. In short, we find that for a zero-priced offer, an increase in incidental costs significantly reduces demand, but for a low-priced offer, an increase in incidental costs does not significantly affect demand. In other words, zero (vs. low) pricing makes consumer demand more sensitive to incidental costs, consistent with the proposition that zero (vs. low) pricing increases the scrutiny of incidental costs.

## Study 1: Field evidence of the boosting and boomerang effects of zero pricing

K-12 after-school tutoring is one of the fastest-growing markets in China in the last decade, with a market size of RMB 800 billion (US $123.7 billion) in 2019 (OliverWyman, 2020). The K-12 education system in China includes 6 years of primary school, 3 years of middle school, and 3 years of high school. After-school tutoring can be provided in offline and online formats, and the formats come with different incidental costs. Specifically, the time cost is relatively high for taking offline classes as students spend a nontrivial amount of time and effort commuting to and from the physical classroom. There is no equivalent incidental cost associated with online classes. According to our hypotheses, a zero (vs. low, nonzero) price should boost demand for online classes (low incidental costs) but hurt demand for offline classes (high incidental costs).

### Method

The data come from one of the largest education companies in China, which offers both online and offline tutoring classes on diverse subjects (e.g., Chinese, English, mathematics) to primary, middle, and high school students. The dataset contains information regarding 3511 classes that were offered from January 2019 to February 2020 in one of the largest cities in central China. The classes were short-term and offered for free or at a discount to promote the company’s long-term classes. For each class, the dataset includes the type of class (online or offline), total fee, duration of each session, number of sessions, enrollment quota, and number of sign-ups, as well as several variables that we include as controls in a supplemental analysis (Web Appendix B).

For the offline classes (high incidental costs), we predicted an inverted-U-shaped relationship between price and demand. Demand generally increases as the price decreases (i.e., the law of demand), but as the price approaches zero, our proposed scrutiny mechanism should cause consumers to consider the high incidental costs of the offline class, and demand should drop (i.e., the hypothesized boomerang effect). Thus, we expected the highest demand for offline classes to occur at a low, nonzero price. For the online classes (low incidental costs), we predicted a linear, negative relationship between the price and consumer demand, such that the highest demand occurs when the price is zero (i.e., the boosting effect).

To test our hypotheses, we first calculated the hourly price of each class, $\frac{total\  fee}{\left( hours\  per\  session\right)\times \left( numbers\ of\ sessions\right)}$. The classes varied in the number of sessions and the duration of each session, so the hourly price enabled us to make valid comparisons across classes. We defined consumer demand for a class as the sign-up rate, $\frac{\  number\ of\ \mathit{\operatorname{sign}}- ups}{quota}$, where the quota is the maximum number of students allowed in the class. The online classes, unconstrained by physical space, had a higher average quota than the offline classes, *M*_*online*_ = 291.48, *SD* = 221.24, *M*_*offline*_ = 39.10, *SD* = 62.40, *F* (1, 3509) = 2418.74, *p* < .001. Unsurprisingly, the average number of sign-ups also was higher for online classes than for offline ones, *M*_*online*_ = 110.90, *SD* = 148.01, *M*_*offline*_ = 23.25, *SD* = 47.39, *F* (1, 3509) = 633.11, *p* < .001. Therefore, we consider the sign-up rate to be a more valid measure of consumer demand than the number of sign-ups, which is influenced by the quota.

The hourly price of each class ranged from CNY ¥0 to ¥174 (approx. US $25.27), *M* = ¥15.58, *SD* = 21.49. Following established conventions (Howell, 1998), we defined outliers as 3 standard deviations from the mean, and we excluded them (*N* = 9), leaving a final dataset of 3502 classes. We regressed the sign-up rate on the linear and quadratic terms of the hourly price, with the class type (1 = online, 0 = offline) as a moderator. To reduce collinearity, we centered the variables in any polynomial for testing interaction effects and quadratic trends (Belsley et al., 1980). If the results indicated a significant moderating effect of the class type on the linear or quadratic term of the hourly price, then we analyzed the data separately for each class type by regressing the sign-up rate on the linear and quadratic terms of the hourly price. Again, the price was centered in the quadratic terms to reduce collinearity.

### Results and discussion

**Fig. 2:**
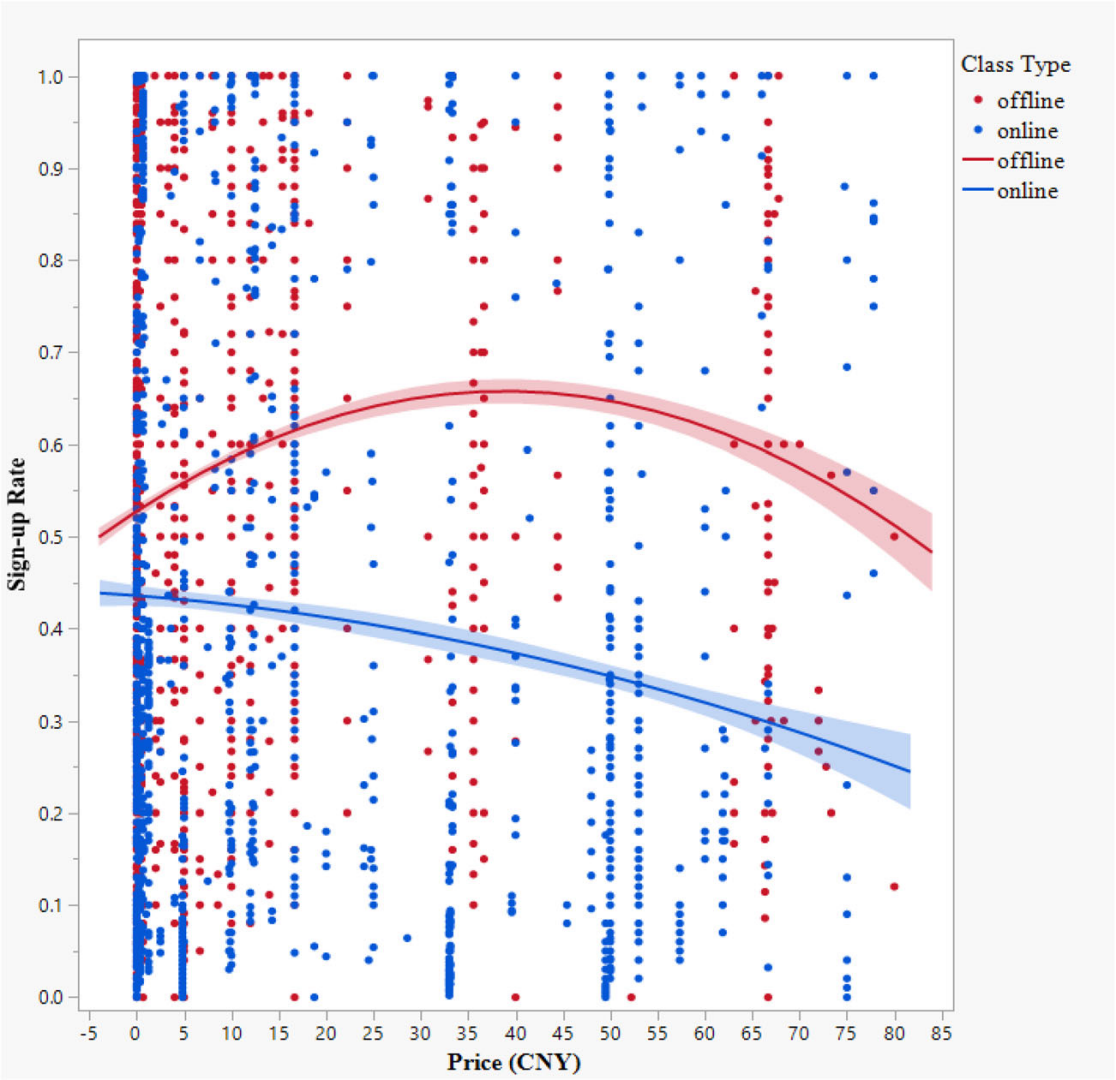
A scatter plot with regression lines for online and offline classes (Study 1)

In the main regression, we found a significant main effect of the class type (1 = online, 0 = offline) such that the sign-up rate was significantly lower for online classes than for offline classes (*b* = −.19, *SE* = .02, *t*(3496) = −10.91, *p* <. 001). More importantly, we found significant linear and nonlinear effects of the hourly price (price: *b* = −.1.82e-3, *SE* = 5.23e-4, *t* (3496) = 3.49, *p* <. 001; price^2^: *b* = −5.85e-5, *SE* = 1.51e-5, *t*(3496) = −3.88, *p* < .001), and both effects were significantly moderated by the class type (price × class type: *b* = −5.43e-3, *SE* = 1.04e-3, *t*(3496) = −5.24, *p* < .001; price^2^ × class type: *b* = 6.72e-5, *SE* = 2.99e-5, *t*(3496) = 2.24, *p* = .025), indicating that the relationship between the hourly price and sign-up rate is different for online and offline classes. As planned, we conducted regression analyses for each type of class. The regression lines appear in Fig. 2.

For online classes, the hourly price has a significant linear term and an insignificant quadratic term (price: *b* = −1.63e-3, *SE* = 5.38e-4, *t*(1435) = −3.03, *p* = .003; price^2^: *b* = −1.89e-5, *SE* = 2.27e-5, *t*(1435) = −0.83, *p* = .405). The negative coefficient of the linear term indicates that an increase in the price significantly reduces the sign-up rate, consistent with our prediction of a linear, negative relationship between the price and demand when incidental costs are relatively low. This supports H1a (the boosting effect of zero pricing).

For offline classes, the hourly price has significant linear and quadratic terms (price: *b* = 4.85e-3, *SE* = 8.52e-4, *t*(2061) = 5.69, *p* < .001; price^2^: *b* = −8.01e-5, *SE* = 2.00e-5, *t*(2061) = −4.31, *p* < .001). The positive coefficient of the linear term indicates that an increase in the price generally increases the sign-up rate for offline classes, while the negative coefficient of the quadratic term indicates that the sign-up rate stops increasing and then falls above a certain price, thus supporting our prediction of an inverted-U-shaped relationship between price and demand when incidental costs are relatively high. This supports H1b (the boomerang effect of zero pricing).

As a robustness check, we repeated the regression with covariates, and the results are qualitatively the same. See Web Appendix B for details.

If zero pricing has a special effect on consumer demand, then we would expect the quadratic relationship between price and demand to vanish when we exclude the 1082 zero-priced classes from the analysis. We ran the same regression model on the remaining 2420 classes. As before, we found a significant main effect of the class type such that the sign-up rate was lower for online classes than for offline classes (*b* = −.27, *SE* = .02, *t*(2414) = −10.06, *p* <. 001). The linear term of the hourly price remained marginally significant (price: *b* = −9.02e-4, *SE* = 4.74e-4, *t*(2414) = −1.90, *p* = .057), but the quadratic term became insignificant (price^2^: *b* = −1.19e-5, *SE* = 1.70e-5, *t*(2414) = −0.70, *p* = .483). The class type significantly moderated the linear term but not the quadratic term of the hourly price (price × class type: *b* = −4.22e-3, *SE* = 9.52e-4, *t*(2414) = −4.44, *p* < .001; price^2^ × class type: *b* = 5.59e-5, *SE* = 3.40e-5, *t*(2414) = 1.64, *p* = .101). We conclude that in the absence of zero-priced classes, the relationship between price and demand is negative and linear, with a steeper slope for online classes than for offline ones. These results align with our contention that the boomerang effect on demand under high incidental costs is special to zero pricing; it does not occur even for the very lowest nonzero prices in the dataset.

The results of Study 1 support our prediction that a zero price boosts demand for a product with low incidental costs, but a low, nonzero price leads to higher demand than a zero price for a product with high incidental costs. In the next study, we use a lab experiment to test the hypothesized dual-process mechanism with explicit measures of positive affect and concerns about incidental costs.

## Study 2: Testing the dual-process model

While Study 1 used a continuous measure of price, Study 2 uses only two price levels: zero and low, nonzero. As in Study 1, the promotional offer is a class, either online or offline. According to our theory, consumers scrutinize incidental costs more in the zero (vs. low) price condition, so an offline class should be less attractive if it is offered for free (vs. for a low price). Meanwhile, for online classes, the positive affect triggered by a zero price is not undermined by concerns about incidental costs (as there are few), so an online class should be more attractive if it is offered for free (vs. for a low price).

### Method

Participants were 205 students (*M*_age_ = 20.59, *SD* = 2.27; 68% female) from a large Midwestern university in the United States. Participants completed an online survey on consumer behavior (in which they evaluated a stress management class, offered either online or offline^1^) for a chance to win a $40 gift card for a major online retailer.

We employed a 2 (price level: zero vs. low) × 2 (class type: offline vs. online) between-subjects design, and participants were assigned randomly. First, all participants read a description of a new “Stress Management” course offered by a clinical center, with the intention of helping college students successfully navigate their academic and social lives. The course would consist of four weekly classes, each lasting 2 h. Participants in the offline-class conditions were told that the classes would be held on another campus; it is common knowledge in the student body that the other campus is about 40 min away (though the commute time was not stated explicitly in the paragraph). Participants in the online-class conditions were told that the classes would be held online.

All participants learned that the first class would be a trial lesson, and they could take it either for free (zero-price conditions) or for only $2 (low-price conditions). Participants indicated their interest in attending the trial lesson on a 7-point scale (“To what extent do you want to attend the trial lesson?” 1 = not at all, 7 = very much), and then they were asked to write down everything that went through their minds when they considered attending.

To measure participants’ affective reactions toward the offer, we adopted the approach used in Shampanier et al. (2007): participants viewed a schematic of seven faces, with expressions ranging from very unhappy to very happy, and chose the face that most closely aligned with their feelings toward the offer. Finally, participants answered demographic questions and were thanked and debriefed.

### Results and discussion

#### Interest in attending the class

**Fig. 3:**
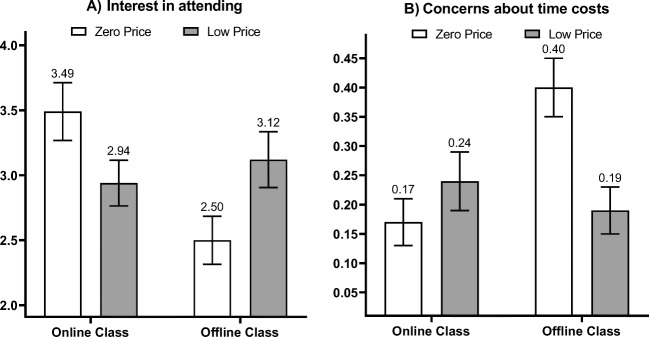
Interest in the offer (**A**) and concerns about time costs (**B**) as functions of the price level and class type (Study 2). (Note: Error bars are standard errors)

A 2 (price level: zero vs. low) × 2 (class type: offline vs. online) ANOVA on the interest in attending the class revealed a significant main effect of the class type; participants in the offline-class condition were less interested in attending than participants in the online-class condition (*M*_offline_ = 2.80, *M*_online_ = 3.20; *F*(1, 201) = 4.17, *p* = .043, ƞ_p_^2^ = .02). The ANOVA found no significant main effect of the price level (*F*(1, 201) = .04, *p* = .852, ƞ_p_^2^ = .00). More importantly, the interaction between the price level and class type was significant (*F*(1, 201) = 8.53, *p* = .004, ƞ_p_^2^ = .04, see Panel A in Fig. 3). For the offline class (high incidental costs), a zero (vs. low) price led to lower interest in attending, consistent with H1b (*M*_zero-price_ = 2.50, *M*_low-price_ = 3.12; *F*(1, 201) = 4.81, *p* = .029, ƞ_p_^2^ = .02); for the online class (low incidental costs), a zero (vs. low) price led to higher interest in attending, consistent with H1a (*M*_zero-price_ = 3.49, *M*_low-price_ = 2.94; *F*(1, 201) = 3.75, *p* = .054, ƞ_p_^2^ = .02). In Web Appendix A, we examine the interaction by testing the impacts of the class type on consumer demand at each price level.

#### Concerns about the time cost and course quality

We used the thought-listing task to investigate whether a zero (vs. low) price raises more concerns about incidental costs, especially time costs, for the offline class than for the online class. A coder blind to the hypotheses and experimental conditions coded each thought as relevant to time costs if the writing mentioned concerns about time and/or convenience. The coder also determined the valence of each thought about time costs (favorable, neutral, or unfavorable) and counted the number of thoughts each participant listed. We operationalized concerns about time costs as a ratio: (number of unfavorable thoughts about time costs - number of favorable thoughts about time costs) / total number of thoughts. The coder used the same method to code thoughts about course quality, and we calculated the analogous ratio for concerns about course quality.

We conducted two 2 (price level: zero vs. low) × 2 (class type: offline vs. online) ANOVAs on concerns about time costs and course quality. Nine participants did not write any thoughts and were excluded from the analysis. The ANOVA on concerns about time costs showed a significant main effect of the class type; concerns about time costs were higher for the offline class (*M*_offline_ = .30, *M*_online_ = .21; *F*(1, 192) = 4.23, *p* = .041, ƞ_p_^2^ = .02). The main effect of the price level was not significant (*F*(1, 192) = 2.68, *p* = .103, ƞ_p_^2^ = .01). More importantly, the interaction between the price level and class type was significant (*F*(1, 192) = 9.44, *p* = .002, ƞ_p_^2^ = .05; see Panel B in Fig. 3). Specifically, the zero (vs. low) price prompted more concerns about time costs for the offline class (*M*_zero-price_ = .40, *M*_low-price_ = .19; *F*(1, 192) = 10.98, *p* = .001, ƞ_p_^2^ = .05) but not for the online class (*M*_zero-price_ = .17, *M*_low-price_ = .24; *F*(1, 192) = 1.04, *p* = .31, ƞ_p_^2^ = .01). Decomposed another way, the interaction indicates that the main effect of the class type on concerns about time costs was driven by the participants who saw a zero price (*M*_offline_ = .40, *M*_online_ = .17, *F*(1, 192) = 13.16, *p* < .001, ƞ_p_^2^ = .06), not by those who saw a low price (*M*_offline_ = .19, *M*_online_ = .24, *F*(1, 192) = .52, *p* = .473, ƞ_p_^2^ = .00). The sensitivity of participants in the zero-price condition to the difference in incidental costs between offline and online classes supports H2b (that a zero price elicits more scrutiny of incidental costs than a low, nonzero price).

The ANOVA on concerns about course quality revealed no significant main or interaction effects (all *p*s > .68). The null results suggest that a zero price does not signal a lower-quality offering than a low price, and online and offline classes do not differ in perceived quality.

#### Positive affect

A 2 (price level: zero vs. low) × 2 (class type: offline vs. online) ANOVA on positive affect in response to the offer showed a significant main effect of the price level: a zero price led to a more positive affect (*M*_zero-price_ = 4.59, *M*_low-price_ = 4.00; *F*(1, 201) = 12.23, *p* < .001, ƞ_p_^2^ = .06), as predicted by H2a and consistent with the finding of Shampanier et al. (2007). The class type did not have a significant main effect on positive affect (*F*(1, 201) = .34, *p* = .563, ƞ_p_^2^ = .00), and there was no significant interaction effect (*F*(1, 201) = .62, *p* = .431, ƞ_p_^2^ = .00).

#### Moderated mediation analysis

We tested the dual-process model using a moderated mediation analysis with the price level as the independent variable, interest in attending the class as the dependent variable, positive affect and concerns about time costs as the mediators, and the class type as the moderator (Model 8 in PROCESS for SPSS, 5000 bootstrap samples; Hayes, 2017).

**Fig. 4:**
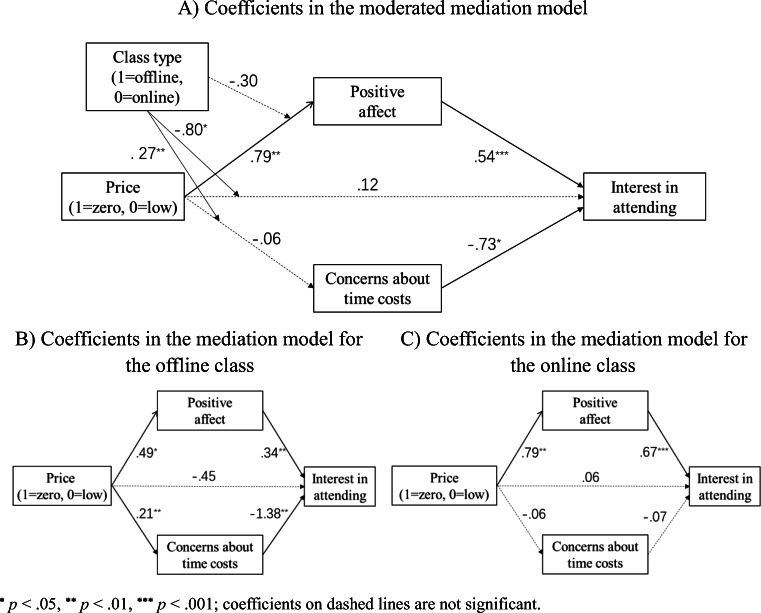
Coefficients of the moderated mediation model (**A**), and coefficients of the mediation models for the offline class (**B**) and online class (**C**) separately (Study 2)

The results showed that positive affect significantly mediated the effect of the zero (vs. low) price on the interest in attending (supporting H2c), irrespective of the class type (*b*_offline_ = .26, *SE* = .12, bias corrected 95% confidence interval (BC 95% CI) [.04, .53]; *b*_online_ = .42, *SE* = .15, BC 95% CI = [.16, .74]). However, the mediation effect through concerns about time costs was significantly moderated by the class type; the mediation effect was significant only for the offline class (*b* = −.15, *SE* = .07, BC 95% CI = [−.34, −.04]), not for the online class (*b* = .05, *SE* = .05, BC 95% CI = [−.03, .18]), supporting H2d. Furthermore, after controlling for the moderated mediation, the interaction effect between the price level and class type diminished, though it remained significant (before^2^: *p* = .005; after: *p* = .031). Figure 4 shows all coefficients in the moderated mediation model and the separate mediation models for offline and online classes.

Study 2 replicated the key finding of Study 1: zero pricing can have both boosting and boomerang effects on demand, and the outcome depends on the incidental costs associated with the offer. Study 2 also tested the mechanisms in the proposed dual-process model. Consistent with the findings in Shampanier et al. (2007), a zero price led to a more positive affect than a low, nonzero price, and a more positive affect enhanced consumer demand. The affective route was the only significant mechanism when incidental costs were low, so the zero price had a boosting effect. Extending the work of Shampanier et al. (2007), we also found that when incidental costs were high, a zero (vs. low) price led to more concerns about incidental costs, and these concerns decreased consumer demand so much that the zero price led to lower consumer demand than the low price (i.e., the boomerang effect).

The results of Study 2 cast doubt on the quality-inferencing mechanism. Theoretically, even if consumers inferred lower quality from a zero price than from a low price, it is not parsimonious or easy to explain why consumers would apply this inference only to the offline class, not the online class. Empirically, the results of Study 2 found null effects of the class type, price level, and their interaction on concerns about course quality. It seems that consumers do not infer quality from the price level or the class type, nor can quality inferences explain the boomerang effect.

Using the thought-listing task, we also examined whether the price level (zero vs. low) has any effect on the total number of thoughts that participants generated. A 2 (price level: zero vs. low) × 2 (class type: offline vs. online) ANOVA on the total number of thoughts showed no significant main or interaction effects (*p*s > .15). Thus, the boomerang effect occurs because zero pricing affects not the *amount* of elaboration but rather the *nature* of elaboration—specifically, the extent to which consumers consider incidental costs.

The present research argues that the boomerang effect of zero pricing occurs because zero pricing facilitates the cognitive scrutiny of incidental costs. In other words, the effect involves a deliberative process. Study 2 supported the deliberative process by showing the mediating role of concerns about incidental costs. Study 3 provides more direct evidence by manipulating participants’ cognitive load.

## Study 3: Confirming the role of scrutiny: Reversal of the boomerang effect under a high cognitive load

### Method

Participants were 306 female students (*M*_age_ = 20.10, *SD* = 2.53) recruited from a large university in China. They received cash payments for participating in a study involving a decision about taking a class on mindfulness. We recruited only female participants because existing literature documents that women are more interested than men in the practice of mindfulness (e.g., Bluth et al., 2017), and women also benefit more than men from the practice of mindfulness (Rojiani et al., 2017).

The study had a 2 (price level: zero vs. low) × 2 (cognitive load: high vs. low) between-subjects design. Unlike Studies 1 and 2, Study 3 included only one type of offer: an offline class in a relatively distant place (i.e., high incidental costs). Study 3 tested whether a high cognitive load can eliminate or even reverse the boomerang effect. Specifically, we predicted that the zero price would trigger the scrutiny of incidental costs in the low-load condition (leading to the boomerang effect of zero pricing) but not in the high-load condition—leading to the boosting effect of zero pricing despite the high incidental costs.

We adopted an existing cognitive load manipulation for college students in Asia (Hong & Sun, 2018), in which participants must memorize a number and then remember it while completing other tasks. Participants in the high-load conditions memorized an 11-digit number (91638742015), while those in the low-load conditions memorized a 3-digit number (916). After participants were instructed to memorize and remember the number, they proceeded to an ostensibly unrelated task about a mindfulness class. Participants read a paragraph of basic information about mindfulness (e.g., its definition) and were informed that a leading institution in the area would launch a series of mindfulness classes soon. Importantly, all participants were told that the series would be held on a different campus of the university. It is common knowledge in the student body that the other campus is about 60 min from the main campus (though the commute time was not stated explicitly in the paragraph). All participants learned that the first class would be a trial lesson, and they could take it either for free (zero-price conditions) or for only ¥5 (approx. US $0.73; low-price conditions).

We included several dependent measures to increase the robustness of the results. Participants indicated their interest in attending the trial lesson on a 7-point scale (“To what extent do you want to attend the trial lesson of this mindfulness course?”; 1 = not at all, 7 = very much) and made a binary choice (“Do you want to attend the trial lesson of this mindfulness course?” yes or no). As a behavioral measure, participants were asked to provide an email address if they wanted to sign up for the class. After completing the dependent measures, participants wrote down the memorized number, for consistency with our cover story. They answered several demographic questions and were thanked and debriefed.

### Results and discussion

#### Interest in attending

A 2 (price level: zero vs. low) × 2 (cognitive load: low vs. high) ANOVA on the interest in attending the mindfulness class revealed no main effects of the price level or cognitive load, *p*s > .60, but the interaction effect was significant (*F*(1, 302) = 11.54, *p* = .001, ƞ_p_^2^ = .04). Among participants with a low cognitive load, the zero price led to a lower interest in attending than the low price, replicating the boomerang effect in Studies 1 and 2 and supporting H1b (*M*_zero-price_ = 3.69, *M*_low-price_ = 4.15; *F*(1, 302) = 4.07, *p* = .045, ƞ_p_^2^ = .01). Among participants with a high cognitive load, however, the effect reversed: the zero price led to a higher interest in attending (*M*_zero-price_ = 4.15, *M*_low-price_ = 3.53; *F*(1, 302) = 7.84, *p* = .005, ƞ_p_^2^ = .03).

#### Binary choice

The results of a generalized linear model reflected an analogous pattern for the binary dependent measure. There were no significant main effects of the price level or cognitive load, *p*s > .97, but the interaction effect was significant, χ^2^(1) = 8.87, *p* = .003. Among participants with a low cognitive load, a smaller proportion of participants indicated that they wanted to attend the class in the zero-price condition than in the low-price condition (*M*_zero-price_ = 31.17%, *M*_low-price_ = 47.89%; χ^2^(1) = 4.35, *p* = .037), supporting H1b. The effect reversed among participants with a high cognitive load (*M*_zero-price_ = 47.62%, *M*_low-price_ = 31.08%; χ^2^(1) = 4.53, *p* = .033).

#### Sign-up

The results of a generalized linear model on the behavioral measure showed the same pattern. There were no significant main effects of the price level or cognitive load, *p*s > .51, but the interaction effect was significant, χ^2^(1) = 9.93, *p* = .002. Among participants with a low cognitive load, a smaller proportion provided an email in the zero-price condition than in the low-price condition (*M*_zero-price_ = 14.29%, *M*_low-price_ = 32.39%; χ^2^(1) = 6.94, *p* = .008), supporting H1b. The effect reversed among participants with a high cognitive load (*M*_zero-price_ = 29.76%, *M*_low-price_ = 7.57%; χ^2^(1) = 3.26, *p* = .071).

As expected, and consistent with Studies 1 and 2, a zero price drove down the demand for an offer with high incidental costs when participants had ample cognitive resources, but a zero price boosted demand when participants had a high cognitive load. Decomposing the interaction effect another way, a high (vs. low) cognitive load increased the attractiveness of the zero-priced offer (interest: 4.15 vs. 3.69, *F*(1, 302) = 4.42, *p* = .036, ƞ_p_^2^ = .01; choice: 47.62% vs. 31.17%, χ^2^(1) = 4.57, *p* = .032; sign-up: 29.76% vs. 14.29%, χ^2^(1) = 5.68, *p* = .017). All results support our argument that the boomerang effect of zero pricing comes from a deliberative process rather than an automatic association of zero pricing with negative concepts such as low quality (e.g., Niemand et al., 2019).

Unexpectedly, a high (vs. low) cognitive load also reduced the attractiveness of the low-priced offer (interest: 3.53 vs. 4.15, *F*(1, 302) = 7.22, *p* = .008, ƞ_p_^2^ = .02; choice: 31.08% vs. 47.89%, χ^2^(1) = 4.35, *p* = .037; sign-up: 7.57% vs. 32.39%, χ^2^(1) = 4.31, *p* = .038). One plausible explanation is that a high cognitive load reduces consumers’ ability to compare the low price with the original price (Mao, 2016); although participants were not informed of the original price in Study 3, they could have made an educated guess based on prior experience. Unless they spend effort on the comparison, consumers may not notice the savings, thus reducing the attractiveness of the low-priced offer.

## Study 4: Replicating the boomerang effect within a single product and testing the specialness of zero pricing

In the first three studies, we assumed that online and offline classes come with low and high incidental costs, respectively. However, online and offline classes may differ on many dimensions other than the time costs of attending, and one may question whether offline classes are distinct from online classes in ways that make a low price more attractive than a zero price. To address this question, Study 4 used the same product (an online seminar) for all participants and took advantage of natural variation in the incidental costs between participants to test the relative effectiveness of zero and low prices. Study 4 also addressed the question of whether the boomerang effect is unique to zero pricing by comparing the effects of a zero price and an extremely low price (1 cent).

All participants received information about an online seminar on resume writing. The seminar required participants to submit a resume draft to an online system before the seminar started, so the incidental costs associated with the offer depended on whether the participant already had a resume available (low effort) or would need to create one from scratch (high effort). We predict that a zero (vs. extremely low) price would boost interest in attending the seminar among participants who already had a resume but would decrease interest among participants who did not.

### Method

Study 4 had a 2 (price level: zero vs. extremely low, manipulated) × 2 (resume availability: yes vs. no, measured) between-subjects design. Participants were 192 students (*M*_age_ = 21.99, *SD* = 1.69; 53% female) recruited from Credamo (www.credamo.com), a data collection platform in China similar to Amazon Mechanical Turk. The recruitment specified that participants needed to be university students and would receive cash payments for completing the study.

In the study, participants were informed of an online seminar that taught resume writing skills for job seekers. The seminar would feature well-known human resources specialists and would be tailored to university students. Participants learned that the seminar was either free (zero-price conditions) or only ¥0.01 (about US $0.0016; extremely-low-price conditions). All participants read that they would need to prepare and upload a draft of their own resume to an online folder before the seminar began so that the speakers could tailor the content of the seminar to address the weaknesses in the uploaded drafts.

Participants indicated their interest in attending the online seminar (“To what extent do you want to attend this seminar on resume writing and interview skills?” 1 = not at all, 7 = very much). On the next page, participants indicated whether they already had a draft of their resume (yes or no). In the end, they answered several demographic questions and were thanked and debriefed.

### Results and discussion

A 2 (price level: zero vs. extremely low) × 2 (resume availability: yes vs. no) ANOVA on the interest in attending the seminar showed no significant main effects of the price level or resume availability (*F*_price_(1, 188) = .46, *p* = .497, ƞ_p_^2^ = .00; *F*_resume_(1, 188) = 2.64, *p* = .106, ƞ_p_^2^ = .01); however, the interaction effect was significant (*F*(1, 188) = 6.21, *p* = .014, ƞ_p_^2^ = .032). Among participants with an available resume, the zero price (vs. an extremely low price) increased interest in the online seminar (*M*_zero_ = 6.09, *M*_extreme-low_ = 5.80; *F*(1, 188) = 2.75, *p* = .099, ƞ_p_^2^ = .01). Among participants without an available resume, however, the zero price reduced interest (*M*_zero_ = 5.44, *M*_extreme-low_ = 5.94; *F*(1, 188) = 3.59, *p* = .060, ƞ_p_^2^ = .02).

Study 4 achieved three objectives. First, the study replicated the key effects (H1a and H1b) with the same product offering, thereby eliminating concerns about potential confounding differences between online and offline classes. Second, by comparing the zero price with an extremely low price, Study 4 suggests that the boomerang effect is indeed special to zero pricing. Third, Study 4 provided additional evidence against the quality-inferencing mechanism, given that the two prices (zero and 1 cent) signal very similar quality.

## Study 5: Generalizing the boomerang effect from high-effort contexts to a high-risk context

Recall that incidental costs have two dimensions: effort and risk (Murphy & Enis, 1986). The first four studies focused on incidental costs that involved more effort than risk (a lengthy commute in Studies 2 and 3; writing a resume in Study 4). By contrast, Study 5 tested whether the boomerang effect occurs with high incidental costs that involve more risk than effort.

Many people are concerned about the effectiveness and safety of vaccines (even though every vaccine undergoes rigorous clinical testing before approval). For new vaccines, concerns about side effects and other risks are especially pronounced (e.g., Henrich & Holmes, 2009) and may lead to slow vaccine uptake, as in the ongoing COVID-19 pandemic. Concerns about the risk associated with a vaccine entail an emotional cost (Anderson et al., 2019); consumers who accept the vaccine despite the risk may worry about potential adverse effects for days (or longer) after vaccination. Because the risk of adverse effects is inherent to vaccines and does not benefit the seller, it qualifies as an incidental cost.

In Study 5, participants decided whether to attend a promotional event for a vaccine that was framed as either well established in the market (low risk) or new to the market (high risk). We expected that the zero (vs. low) price would be more effective for promoting the vaccine in the low-risk condition but would be less effective in the high-risk condition.

### Method

Participants were 376 college students (*M*_age_ = 23.58, *SD* = 2.72; 42% female) recruited from the same university as the participants in Study 3. They received cash payments for participating in the study.

Participants were randomly assigned within a 2 (price level: zero vs. low) × 2 (nonmonetary risk: high vs. low) between-subjects design. All participants read a paragraph with basic information about hepatitis C. Participants were informed that a pharmaceutical company had developed a vaccine for hepatitis C, and the vaccine had demonstrated clinical effectiveness. Participants in the low-risk conditions were told that the vaccine had been widely used in the market for a while, and it was performing as expected. Participants in the high-risk conditions were told that the vaccine was not yet widely used in the market, so regulatory authorities were still monitoring its performance closely.

Then, participants learned that the pharmaceutical company was collaborating with a grade A tertiary hospital in Shanghai to promote the vaccine. If participants attended the event, they could get vaccinated either for free (zero-price condition) or for only ¥5 (approx. US $0.73; low-price condition). Participants indicated their interest in attending the promotional event on a 7-point scale (“To what extent do you want to attend this vaccination event?” 1 = not at all, 7 = very much) and made a binary choice (“Do you want to attend this vaccination event?” yes or no).

After participants completed the main dependent measures, they indicated their skepticism of the company’s motive on a scale adapted from Biswas et al. (2013; e.g., “I think the pharmaceutical company uses low prices as a trick to attract people,” *α* = .78; see Web Appendix C for details). Finally, participants answered some demographic questions and were thanked and debriefed.

### Results and discussion

#### Interest in attending

A 2 (price level: zero vs. low) × 2 (nonmonetary risk: high vs. low) ANOVA on the interest in attending the event showed a significant main effect of the nonmonetary risk; participants in the high-risk condition were less interested than participants in the low-risk condition in attending the event (*M*_high-risk_ = 3.62, *M*_low-risk_ = 4.18; *F*(1, 372) = 9.24, *p* = .003, ƞ_p_^2^ = .02). The main effect of the price level was not significant, *p* = .986. Most importantly, there was a significant interaction effect (*F*(1, 372) = 7.60, *p* = .006, ƞ_p_^2^ = .02): When the risk was high, participants in the zero-price condition were less interested in attending than participants in the low-price condition, consistent with H1b (*M*_zero-price_ = 3.37, *M*_low-price_ = 3.87; *F*(1, 372) = 3.75, *p* = .054, ƞ_p_^2^ = .01). When the risk was low, however, participants in the zero-price condition were more interested, consistent with H1a (*M*_zero-price_ = 4.44, *M*_low-price_ = 3.93; *F*(1, 372) = 3.85, *p* = .051, ƞ_p_^2^ = .01).

#### Binary choice

A generalized linear model on the binary measure showed the same patterns. There was a significant main effect of the nonmonetary risk such that a smaller proportion of participants in the high-risk conditions than in the low-risk conditions wanted to attend the vaccination event (*M*_high-risk_ = 40.96% vs. *M*_low-risk_ = 60.64%; χ^2^(1) = 15.24, *p* < .001). The main effect of the price level was not significant (*p* = .732). Critically, there was a significant interaction effect (χ^2^(1) = 8.36, *p* = .004): When the risk was high, a larger proportion of participants in the low-price condition than in the zero-price condition wanted to attend the event (*M*_low-price_ = 47.37%, *M*_zero-price_ = 34.41%; χ2(1) = 3.28, *p* = .070). When the risk was low, the effect reversed (*M*_zero-price_ = 68.82%, *M*_low-price_ = 52.63%; χ^2^(1) = 5.19, *p* = .023).

#### Skepticism

A 2 (price level: zero vs. low) × 2 (nonmonetary risk: high vs. low) ANOVA on skepticism about the company’s motive showed no significant main effects or interaction effect, *p*s >. 16, suggesting that a zero price does not seem less trustworthy than a low price. The null effect of the price level on skepticism casts doubt on the alternative account based on the quality inference.

Study 5 extends the results of Studies 1–4 by demonstrating the boomerang (boosting) effect under high (low) incidental costs that involve *risk* rather than *effort*. Managers should be aware of the potential downside of zero pricing in risky consumption domains as well as domains that involve high effort. Functional risk can accompany all purchases, but especially those that are newer in the market. Our results suggest that managers may need to use different promotional prices at different stages of a product’s life cycle.

## General discussion

### Theoretical contribution

Zero-pricing is a popular strategy in marketing practice and also draws a lot of attention from academia. Most scholars have focused on the positive effects of zero pricing on consumer demand (e.g., Shampanier et al., 2007), while few studies have documented negative effects (e.g., Kamins et al., 2009). As far as we know, no extant research has demonstrated the pros and cons of zero pricing side by side. The current research demonstrates that a zero (vs. low, nonzero) price has a boosting effect on consumer demand when incidental costs are low but has a boomerang effect on demand when incidental costs are high. Our unified theoretical account explains the diverging effects: the boosting effect is driven by the greater positive affect evoked by a zero (vs. low) price, while the boomerang effect occurs because a zero price triggers the cognitive scrutiny of incidental costs, and concerns about high incidental costs can override the affective pathway.

The boomerang effect involves a consumer reaction that is unique to zero prices; the effect does not occur with extremely low prices (including 1 cent). Extant literature has identified several other features that make zero prices “special” (e.g., Shampanier et al., 2007; Mao, 2016). Only a zero price, not a low price, boosts demand by increasing positive affect (Baumbach, 2016; Ma et al., 2018; Shampanier et al., 2007). Also, only a zero price inhibits a comparison of the discounted price against the regular price (Mao, 2016). The present research enriches the literature on zero pricing by identifying another special feature: only a zero price triggers scrutiny of incidental costs. Because incidental costs are implicit, consumers often fail to consider them. In Studies 2, 4, and 5, consumers who were offered a zero price—but not those offered a low price—displayed sensitivity to variation in incidental costs (see Web Appendix A).

Finally, this research underscores the value of a dual-process approach for understanding consumers’ reactions to zero prices. The model provides simultaneous, compatible explanations for the classic boosting effect and the novel boomerang effect. Interestingly, in Study 2, even the consumers with high incidental costs experienced more positive affect toward the zero-priced offer than toward the low-priced offer, though consumers ultimately followed their cognitive reaction over their affective reaction. Thus, zero pricing appears to trigger independent, opposing effects under high incidental costs: a positive affective reaction and a negative scrutiny reaction. This exemplifies a recurring empirical pattern in conceptual analyses of dual-process approaches to understanding attitudes (see Gawronski & Bodenhausen, 2006, “Case 5″). Given the coexistence of conflicting affective and cognitive reactions, consumers who face nontrivial incidental costs may be ambivalent about zero-priced offers.

### Managerial implications

The findings in the present research might help marketers by answering three important practical questions. First, *when* should marketers adopt zero pricing? According to our results, zero pricing is most effective when the typical prospective consumer will face low incidental costs (e.g., commute time and convenience) to obtain/use the offering. Conversely, zero pricing should be avoided when incidental costs are high. In omni-channel pricing, for example, marketing practitioners may benefit from using different promotional pricing strategies for online and offline marketing channels. Consumers face higher incidental costs in the offline channel (e.g., the commute time, shopping time, and, at the time of writing, the risk of exposure to COVID-19), so zero pricing may be less effective than low pricing for product promotions in the offline channel.

Second, to *whom* should marketers target zero-price promotions? Consumers may face heterogeneous incidental costs for the same offering, as in Study 4—the incidental cost of an online resume-writing seminar depended on whether the student had already drafted a resume. Similarly, for an offline class, students may need to travel different distances and via different modes of transportation, so they may incur a range of incidental costs. Therefore, marketers may find it useful to segment consumers according to incidental costs and use the most appropriate pricing strategy for each group.

Third, *how* can marketers increase the effectiveness of zero pricing for boosting demand? The results of Study 3 suggest that marketers can mitigate the boomerang effect by keeping consumers cognitively busy. Of course, socially responsible marketers need to consider consumer welfare when deciding whether and how to increase the cognitive load.

### Limitations and directions for future research

Overall, this is the first work to show that zero (vs. low, nonzero) pricing can hurt consumer demand under specific circumstances. The boomerang effect raises many interesting questions that can be explored in future work. For example, how does zero (vs. low, nonzero) pricing affect the repurchase rate after the promotion ends? We speculate that zero pricing might lead to a higher repurchase rate because consumers who decide to try the product in spite of the incidental costs should have stronger interest in the product. Moreover, public health officials are interested in understanding the factors that influence vaccine uptake. We found a significant interaction between the price level and risk, but we did not manipulate effort; future research might also consider the interaction between the price level and the time cost (e.g., getting vaccinated at one’s local pharmacy vs. at a distant mass vaccination event).

Another fruitful direction for future research would be to investigate the specific mechanisms through which zero prices enhance scrutiny, and in particular, why the mechanisms differ for low, nonzero prices. Perhaps a zero price triggers special curiosity about the offer. Substantial literature shows that zero has a special status in the number system and occupies a special place in cognition (for a review, see Nieder, 2016). For example, children understand the meaning of small, nonzero numbers much earlier than they understand the meaning of zero (e.g., Wellman & Miller, 1986), and zero emerged much later than other numbers in Western civilization (Barrow, 2001; Boyer, 1944). Zero, representing the absence of something, is psychologically distinct from representations of the presence (even a very small presence) of something. Research on the specialness of zero prices upholds the notion that zero is psychologically distinct. Although more precise specifications of the psychological mechanisms may not be crucial for managerial purposes, they could inform more nuanced theoretical development.

## Supplementary Information

ESM 1 (DOCX 196 kb)

## Footnotes

1 Study 2 was conducted before the COVID-19 pandemic.

2 The *p* value was estimated based on the sample that excluded the 9 participants who did not list any thoughts, so the *p* value here is slightly different from the *p* value estimated on the whole sample, *p* = .004.
