# Supplementary material for: The boomerang effect of zero pricing: when and why a zero price is less effective than a low price for enhancing consumer demand
Source: J Acad Mark Sci. 2022 Feb 14;50(3):521–37. doi: 10.1007/s11747-022-00842-1 (PMC8852885; doi:10.1007/s11747-022-00842-1)
Supplement: Supplementary file 1 — (DOCX 196 kb) [file 11747_2022_842_MOESM1_ESM.docx]

Web Appendices of

**The Boomerang Effect of Zero Pricing: When and Why a Zero Price is Less Effective than a Low Price for Enhancing Consumer Demand**

Web Appendix A: The impacts of incidental costs (high vs. low) on consumer demand at each price level2

Web Appendix B: Regression results with control variables in study 13

Web Appendix C: Stimuli and measures in studies 2–55

**Web Appendix A: The impacts of incidental costs (high vs. low) on consumer demand at each price level**

**Table 1** Results of consumer demand in studies 2, 4, and 5

|  |  | High  incidental costs | Low  incidental costs | Contrast: high vs. low incidental costs |
| --- | --- | --- | --- | --- |
| Study 2 | Zero price | 2.50 (1.34) | 3.49 (1.56) | *F*(1, 201) = 12.14, *p* = .001, ƞ_p_^2^ = .06 |
|  | Low price | 3.12 (1.52) | 2.94 (1.29) | *F*(1, 201) = .39, *p* = .532, ƞ_p_^2^ = .00 |
| Study 4 | Zero price | 5.44 (1.26) | 6.09 (0.86) | *F*(1, 188) = 7.77, *p* = .006, ƞ_p_^2^ = .04 |
|  | Low price | 5.94 (1.12) | 5.80 (0.95) | *F*(1, 188) = .41, *p* = .521, ƞ_p_^2^ = .00 |
| Study 5 | Zero price | 3.37 (1.68) | 4.44 (1.80) | *F*(1, 372) = 16.62, *p* < .001, ƞ_p_^2^ = .04; |
|  |  | 34.41% | 68.82% | χ^2^(1) = 22.50, *p* < .001 |
|  | Low price | 3.87 (1.96) | 3.93 (1.75) | *F*(1, 372) = .041, *p* = .840, ƞ_p_^2^ = .00 |
|  |  | 47.37% | 52.63% | χ^2^(1) = .53, *p* = .468 |
| Note: Means and standard deviations (in parentheses) reflect participants’ interest in accepting the offer (studies 2, 4, and 5); the percentages in study 5 reflect the proportions of participants who chose to accept the offer. | | | | |

**Web Appendix B: Regression results with control variables in study 1**

We regressed the sign-up rate on the linear and quadratic terms of the hourly price, with the class type (1 = online, 0 = offline) as a moderator. As covariates, we added the subject matter (main subjects [Chinese, English, and mathematics] or other subjects), school type (primary, middle, or high school), the days the class met (weekdays, holidays [national holidays, weekends, and summer/winter vacation], or mixed [both weekdays and holidays]), and the time of day (morning, afternoon, evening, or mixed). For each covariate, we included both the main effect and interaction effect with the class type. The class type and covariates all are coded as dummy variables. See Table 2 for the results.

Table 2 Regression results with control variables (study 1)

|  |  | Model for All Classes | | | | Model for Online Classes | | | | Model for Offline Classes | | | |
| --- | --- | --- | --- | --- | --- | --- | --- | --- | --- | --- | --- | --- | --- |
|  |  | *b* | *(SE)* | t |  | *b* | *(SE)* | *t* |  | *b* | *(SE)* | *t* |  |
| Price | | .0026 | (.0005) | 4.76 | ^***^ | -.0020 | (.0005) | -3.64 | ^***^ | .0064 | (.0009) | 7.16 | ^***^ |
| Price^2^ | | -7.35e-5 | (1.48e-5) | -4.98 | ^***^ | -3.32e-5 | (2.21e-5) | -1.51 |  | -.0001 | (1.96e-5) | -5.17 | ^***^ |
| Online class | | -.1589 | (.0182) | -8.72 | ^***^ |  |  |  |  |  |  |  |  |
| Online class × Price | | -.0069 | (.0011) | -6.53 | ^***^ |  |  |  |  |  |  |  |  |
| Online class × Price^2^ | | 6.84e-5 | (2.93e-5) | 2.33 | ^*^ |  |  |  |  |  |  |  |  |
| Main subject | | .1573 | (.0144) | 10.96 | ^***^ | .0841 | (.0213) | 3.95 | ^***^ | .2082 | (.0192) | 10.85 | ^***^ |
| Middle school | | -.0042 | (.0144) | -.29 |  | .1128 | (.0253) | 4.45 | ^***^ | -.0857 | (.0171) | -5.02 | ^***^ |
| High school | | -.1809 | (.0224) | -8.09 | ^***^ | -.1794 | (.0337) | -5.33 | ^***^ | -.1819 | (.0297) | -6.13 | ^***^ |
| Holiday | | .0463 | (.0137) | 3.37 | ^***^ | .0570 | (.0214) | 2.67 | ^**^ | .0388 | (.0179) | 2.17 | ^*^ |
| Mixed day | | -.0069 | (.0610) | -.11 |  | -.0747 | (.0404) | -1.85 | ^+^ | .0404 | (.0975) | .41 |  |
| Afternoon | | -.0281 | (.0166) | -1.7 | ^+^ | -.0306 | (.0304) | -1.00 |  | -.0264 | (.0188) | -1.41 |  |
| Evening | | .0385 | (.0169) | 2.28 | ^*^ | .0184 | (.0289) | .64 |  | .0525 | (.0206) | 2.55 | ^*^ |
| Mixed time | | -.1532 | (.1086) | -1.41 |  | -.0507 | (.0626) | -.81 |  | -.2245 | (.1750) | -1.28 |  |
| Online class × Main subject | | -.1241 | (.0285) | -4.36 | ^***^ |  |  |  |  |  |  |  |  |
| Online class × Middle school | | .1985 | (.0301) | 6.6 | ^***^ |  |  |  |  |  |  |  |  |
| Online class × High school | | .0025 | (.0446) | .06 |  |  |  |  |  |  |  |  |  |
| Online class × Holiday | | .0183 | (.0276) | .66 |  |  |  |  |  |  |  |  |  |
| Online class × Mixed day | | -.1151 | (.1073) | -1.07 |  |  |  |  |  |  |  |  |  |
| Online class × Afternoon | | -.0041 | (.0352) | -.12 |  |  |  |  |  |  |  |  |  |
| Online class × Evening | | -.0341 | (.0350) | -.97 |  |  |  |  |  |  |  |  |  |
| Online class × Mixed time | | .1738 | (.1893) | .92 |  |  |  |  |  |  |  |  |  |
| *Observations* | | 3502 | | | | 1438 | | | | 2064 | | | |
| *R^2^* | | .2139 | | | | .1769 | | | | .1606 | | | |
| ^+^ *p* < .10, ^*^ *p* < .05, ^**^ *p* < .01, ^***^ *p* < .001 | | | | | | | | | | | | | |

**Web Appendix C: Stimuli and measures in studies 2–5**

**Study 2**

*Stimuli*

Stress Management and Success Strategies

Life as a NU student offers a dizzying array of challenges and opportunities. The task of balancing academic, social, and extracurricular responsibilities can be stressful. And while stress is a fact of life, too much stress can cause problems. It can impede your cognitive abilities, mental and physical health and negatively impact your relationships with friends and family. Therefore, the ability to effectively manage stress is an important life skill.

The Osher Center for Integrative Medicine will offer a session of classes on Stress Management and Success Strategies in May and June. The classes will provide you opportunities to identify physical and emotional symptoms that let you know when you are under stress, and learn strategies and techniques (e.g., biofeedback and relaxation) to help you manage the stress, study more efficiently and effectively, and be more healthy in mind, body, and spirit.

*Condition 1 (offline class, zero price)*

The session contains four weekly classes, and each class will last two hours. *The classes will be held in a building on the Chicago campus*. The first week class will be a trial lesson. There is a promotional price of the trial lesson: People who are interested in the classes can take the trial lesson *for free*.

OR

*Condition 2 (offline class, low price)*

**The session contains four weekly classes, and each class will last two hours. *The classes will be held in a building on the Chicago campus*. The first week class will be a trial lesson. There is a promotional price of the trial lesson: People who are interested in the classes can take the trial lesson by *paying $2*.**

OR

*Condition 3 (online class, zero price)*

**The session contains four weekly classes, and each class will last two hours. *The classes will be given online. Both the live classes and recorded videos are available to registered members.* The first week class will be a trial lesson. There is a promotional price of the trial lesson: People who are interested in the classes can take the trial lesson *for free*.**

OR

*Condition 4 (online class, low price)*

**The session contains four weekly classes, and each class will last two hours. *The classes will be given online. Both the live classes and recorded videos are available to registered members.* The first week class will be a trial lesson. There is a promotional price of the trial lesson: People who are interested in the classes can take the trial lesson by *paying $2*.**

Please click “>>” after you read the above information. We will ask you questions regarding your reactions toward the trial lesson.

*Measure of consumer demand*

Regarding this session of classes on Stress Management and Success Strategies......

To what extent do you want to attend the trial lesson? (1 = not at all, 7 = very much)

*Thought-listing task*

We are interested in everything that went through your mind when you made the decision of attending or not attending the free ($2) trial lesson. Please list all your thoughts below, whether they were about the classes, the trial lesson, and/or others; whether they were positive, negative, and/or neutral thoughts. We have deliberately provided more space than we think people will need, to ensure that everyone would have plenty of room.

*Positive affect*

Overall, how do you feel about this offer of taking the trial lesson for free (by paying $2)?


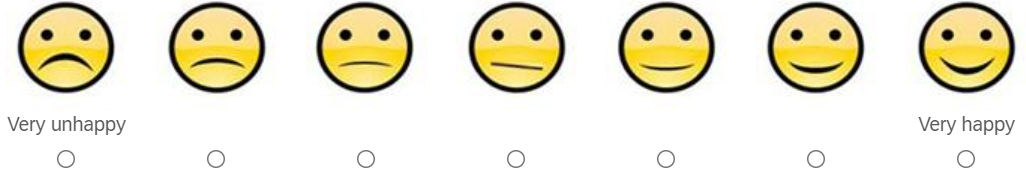


**Study 3**

*Stimuli (originally in Chinese)*

Part I: Memory test

*High-cognitive-load condition*

Please remember the following *11*-digit number. Do not use paper and pen or a mobile phone to record the number. Do your best to remember it, and we will test your memory of it at a later time.

*91638742015*

OR

*Low-cognitive-load condition*

Please remember the following *3*-digit number. Do not use paper and pen or a mobile phone to record the number. Do your best to remember it, and we will test your memory of it at a later time.

*916*

Part II: A survey about a mindfulness class

In this session, you will see a survey about taking a trial lesson on practicing mindfulness. As a reminder, when you read and answer questions, please do not forget the number presented on the previous page.

A survey about your inclination to attend a trial lesson on practicing mindfulness

We live in a busy and noisy world, and our lives are full of plans, conflicts, deadlines, exams, and performance evaluations. Research shows that adults are distracted 47% of the time due to various pressures. Disorganized minds have many negative effects on learning, living, family, work, and other aspects. How can we regain peace, self-healing, control, and happiness in a complex, modern society? We encourage you to practice mindfulness, a physical and mental training method that is endorsed by and widely used in modern Western society.

Mindfulness is the practice of purposely bringing one’s attention to the present moment, without judgment and with full attention to your current physical and mental experience. The practice of mindfulness was developed by Dr. Kabat-Zinn from the Massachusetts Institute of Technology. He created this practice based on the results of research in medicine, neuroscience, and Eastern meditation philosophy. Nowadays, large companies and business schools from Wall Street to Silicon Valley are providing mindfulness programs for their employees. In addition, mindfulness is also widely used in sports training, the mental rehabilitation of veterans, and education. The results of experiments in both companies and schools have shown that practicing mindfulness can change the function and structure of the brain, improve concentration, enhance creativity, improve decision-making, reduce stress and anxiety, reduce mood swings, improve interpersonal communication, improve sleep, and enhance immune function.

The California Institute of Health (CIH) is one of China’s leading research and teaching institutions on the topic of mindfulness. CIH is launching a four-week mindfulness course for college students from December this year to January next year in Shanghai. The course is taught by a well-known mindfulness educator at CIH. Classes will be taught on the Xuhui campus of Shanghai Jiao Tong University. Each class lasts for two and a half hours. The first class is a trial lesson. CIH is providing a discount for the trial lesson:

*Zero-price condition*

participants can take the trial lesson for *free*.

OR

*Low-price condition*

participants can take the trial lesson for *only ¥5*.

We are helping CIH investigate students’ interest in attending the trial lesson. If you have read the above information carefully, please proceed to the next page to answer questions.

*Measures of consumer demand*

- Do you want to attend the trial lesson of this mindfulness course? (Yes, No)
- To what extent do you want to attend the trial lesson of this mindfulness course? (1 = not at all, 7 = very much)
- If you want to sign up for the trial lesson of this mindfulness course, please enter your email address in the space below. We will coordinate the classroom according to the number of attendees and will inform the attendees of the specific location and time of the trial lesson by email.

Please enter the number that you were asked to remember in Part I:

**Study 4**

*Stimuli (originally in Chinese)*

Job Hunting Seminar: Resume Writing and Interview Skills

The resume is an essential element for job hunting and applying for an advanced degree program. According to the US Department of Labor, the average HR employee spends only 15 seconds reading each resume. How do you write a resume that is so concise and impressive that it can earn you a new opportunity in just 15 seconds?

NetEase Cloud Classroom is inviting well-known human resources specialists to give an online seminar that teaches resume writing and interview skills.

*Zero-price condition*

NetEase Cloud Classroom provides discounts for college students. College students can attend the online seminar for *free*.

OR

*Extremely-low-price condition*

NetEase Cloud Classroom provides discounts for college students. College students can attend this online seminar for *only 1 cent*.

Seminar attendees must prepare a personal resume and upload it to the Cloud Classroom Mailbox for this lecture. Only those who finish the task can attend the seminar. The speakers will customize the content of the seminar to address the weaknesses in the uploaded resumes.

We are helping NetEase Cloud Classroom investigate students’ interest in the seminar. If you have read the above information carefully, please proceed to the next page to answer questions.

*Measure of consumer demand*

To what extent do you want to attend this seminar on resume writing and interview skills? (1 = not at all, 7 = very much)

*Resume availability*

Do you already have a resume? (Yes, No)

**Study 5**

*Stimuli (originally in Chinese)*

A Survey about the Inclination to Take a Hepatitis C Vaccine

Many people are familiar with the hepatitis A virus and hepatitis B virus. Both viruses can seriously damage the function of the human liver, so many people get vaccines to prevent infections with the two viruses. However, clinical practitioners have recently discovered a new hepatitis virus: hepatitis C. Nearly 15 million people are infected with the hepatitis C virus in our country. Because this new virus spreads quickly and has a long incubation period, experts estimate that in the future, the mortality rate caused by the hepatitis C virus will be much higher than that of types A and B.

The hepatitis C virus usually is transmitted through blood. It does not usually attack the body immediately after infection; its incubation period in the human body can be as long as several years. In daily life, we may be infected with this virus on many types of occasions: touching undressed wounds, kissing, sexual activity, donating blood, and sharing toothbrushes, cups, razors, and nail clippers and files with others.

Given the highly infectious and harmful nature of this new virus, a US pharmaceutical company has developed a vaccine against it. Clinical evidence indicates that the vaccine is effective at reducing the transmission of the hepatitis C virus.

*Condition 1 (high risk, zero price)*

*However, the vaccine is not yet widely used in the market, so its performance is still monitored closely.* The pharmaceutical company is collaborating with a grade A tertiary hospital in Shanghai to promote the vaccine. Despite the high cost of the vaccine R&D process, citizens who sign up for the promotional event can get this vaccine for the hepatitis C virus *for free*.

OR

*Condition 2 (high risk, low price)*

*However, the vaccine is not yet widely used in the market, so its performance is still monitored closely.* The pharmaceutical company is collaborating with a grade A tertiary hospital in Shanghai to promote the vaccine. Despite the high cost of the vaccine R&D process, citizens who sign up for the promotional event can get this vaccine for the hepatitis C virus *for only ¥5*.

OR

*Condition 3 (low risk, zero price)*

*The vaccine is widely used in the market, and it is performing as well as expected.* The pharmaceutical company is collaborating with a grade A tertiary hospital in Shanghai to promote the vaccine. Despite the high cost of the vaccine R&D process, citizens who sign up for the promotional event can get this vaccine for the hepatitis C virus *for free*.

OR

*Condition 4 (low risk, low price)*

*The vaccine is widely used in the market, and it is performing as well as expected.* The pharmaceutical company is collaborating with a grade A tertiary hospital in Shanghai to promote the vaccine. Despite the high cost of the vaccine R&D process, citizens who sign up for the promotional event can get this vaccine for the hepatitis C virus *for only ¥5*.

We are helping them investigate college students’ interest in attending this event.

If you have read the above information carefully, please proceed to the next page to answer questions.

*Measures of consumer demand*

- Do you want to attend this vaccination event? (Yes, No)
- To what extent do you want to attend this vaccination event? (1 = not at all, 7 = very much)

*Skepticism of the company’s motive (**α = .78)*

Based on your thoughts and feelings when making the decision, how much do you agree or disagree with each of the following statements? (1= strongly disagree, 7 = strongly agree)

- I think the pharmaceutical company advertises low prices simply to attract people.
- I think the pharmaceutical company uses low prices as a trick to attract people.
- I think the pharmaceutical company is trying to take advantage of people’s preferences for cheap goods.
